# Temporomandibular disorder as risk factor for radiation‐induced trismus in patients with head and neck cancer

**DOI:** 10.1002/cre2.511

**Published:** 2021-11-10

**Authors:** Nina Pauli, Bodil Fagerberg Mohlin, Christina Mejersjö, Caterina Finizia

**Affiliations:** ^1^ Department of Otorhinolaryngology, Institute of Clinical Sciences Sahlgrenska Academy at the University of Gothenburg, Sahlgrenska University Hospital Gothenburg Sweden; ^2^ Department of Oral and Maxillofacial Surgery, Institute of Clinical Sciences Sahlgrenska Academy at the University of Gothenburg Gothenburg Sweden; ^3^ Clinic of Orofacial Pain Sahlgrenska Academy and Public Dental Health Gothenburg Sweden

**Keywords:** head and neck cancer, pain, radiotherapy, temporomandibular disorder

## Abstract

**Objectives:**

The aim of this study was to investigate if patients with temporomandibular disorders (TMD) prior to head and neck cancer (HNC) treatment are at higher risk of developing trismus after oncological treatment.

**Materials and Methods:**

Eighty‐three study patients underwent detailed dental examination prior oncological treatment and 6 months after radiotherapy completion, including evaluation of temporomandibular jaw function, palpation of the jaw muscles, and measurement of mouth opening capacity. TMD criteria were based on both clinical examination findings and patient‐reported symptoms. TMD at baseline was used in regression analysis in order to predict restricted mouth opening.

**Results:**

At the 6 months follow‐up more than a third of the patients (35%) were examined with reduced mouth opening of >20% compared to baseline. A majority of the patients had bilateral tenderness of both the temporal and the masseter muscle. At the 6 months follow‐up, 42% of the study patients reported mouth opening problems. About one‐third of the patients suffered from fatigue and stiffness of the jaw as well as pain when mowing the jaw. Two questions from the validated symptom‐specific Gothenburg Trismus Questionnaire were found to be related to a statistically significant increased risk of restricted mouth opening after radiotherapy; “During the last week, have you felt: Pain on moving the jaw?” (OR [95% CI] 5.9 (1.2–29.4) [*p* = 0.030]) and “During the last week, have you felt pain and tenderness in the muscles of mastication?” (OR [95% CI] 5.90 [1.19–29.40] [*p* = 0.030]).

**Conclusions:**

TMD is common amongst HNC patients. Patients who suffer from pain in the jaw muscles and pain when moving the jaw before start of treatment are at higher risk of radiation‐induced trismus after 6 months. Clinicians should strive for optimizing the patients pain treatment and oral health before, during, and after radiotherapy.

## 1 BACKGROUND

The masticatory system in humans is strikingly complex and dependent on the function of all its components; bone, muscles, ligaments, joints, vascular supply, and innervation. Disturbances in any of these structures can result in pain and dysfunction for the patient. Temporomandibular disorder (TMD) includes all functional disturbances in the masticatory system (Okeson, 2013).

Most patients with head and neck cancer (HNC) are treated with external beam radiotherapy (RT). Even though RT has developed over the last decades, with higher precision and less toxicity, the treatment‐related side effects remain a heavy symptom burden for patients both short as well as long term (Grégoire et al., 2015). In HNC, the most commonly reported side effects after RT are dry mouth, dysphagia, and restricted mouth opening. These symptoms are known to affect the patients' quality of life severely and persist for a long time (Abel et al., 2020; Langendijk et al., 2008). For restricted mouth opening or trismus, earlier studies have shown that tumor location, tumor size, and RT dose are related to the risk of developing restricted mouth opening after RT (Pauli et al., 2013; van der Geer et al., 2016). The masticatory system is generally affected by the RT to some extent since it is included in the radiation field. It has been hypothesized that certain parts of the masticatory system such as the masseter muscles and the pterygoids are more critical for the development of radiation‐induced trismus than other structures (Teguh et al., 2008; van Der Molen et al., 2013). In the general population, TMD is very common, with some authors reporting up to 60% of the population being affected (Kohler et al., 2013; Lovgren et al., 2016). The question is if patients with TMD have a higher risk of radiation‐induced side effects compared to those without prior symptoms. To our knowledge, there are no earlier studies investigating the role of TMD in HNC patients before RT and the patient's risk of developing restricted mouth opening after RT.

## 2 OBJECTIVES

This study aimed to investigate if patients with TMD prior to HNC treatment are at higher risk of developing trismus after oncological treatment compared to patients without signs of TMD as well as to identify risk factors for trismus development.

## 3 MATERIAL AND METHODS

### 3.1 Patient characteristics and study protocol

Patients with newly diagnosed HNC were invited to participate in the study at the weekly multidisciplinary tumor board conference. The study was carried out at a tertiary referral center in the western region of Sweden and patients were enrolled between 2007 and 2012.

All study patients underwent detailed dental examination prior to the oncological treatment by a specialist in Oral and Maxillofacial surgery (author B.F.M.) according to the study protocol. The study protocol included evaluation of temporomandibular jaw (TMJ) function, measurement of mouth opening (maximal interincisal opening [MIO]), laterotrution capacity, horizontal overbite, vertical overbite, maximal protrusion capacity, mouth opening deviation, TMJ palpation (tenderness and lateralization), TMJ sounds or crepitation and, palpation of the jaw muscles assessing tenderness. Patients were assessed with regards to comorbidities according to adult comorbidity evaluation (ACE‐27) (Paleri et al., 2010). The patients answered the validated symptom‐specific trismus questionnaire Gothenburg Trismus Questionnaire (GTQ) (Johnson et al., 2012). The GTQ contains items regarding jaw‐related problems, pain in face and jaw, trismus, and its impact on quality of life and daily activities. Patients were assessed before oncological treatment and at 6 months after completed treatment.

Inclusion criteria were: newly diagnosed HNC (ICD codes: C01‐C11) receiving oncological treatment (RT ± chemotherapy) and age >18 years. Exclusion criteria were: surgical treatment only, trismus at baseline (MIO ≤35 mm), recurrent disease, poor general health, difficulties in filling out questionnaires, and edentulous patients. Furthermore, patients with tumors treated with RT where the muscles of mastication were not included in the radiation fields were excluded.

### 3.2 Endpoints and TMD criteria

TMD criteria were based on both clinical examination findings and patient‐reported symptoms. For TMD diagnosis according to clinical examination, the presence of restricted mouth opening (MIO ≤35 mm) or tenderness in the temporomandibular joint or muscular tenderness in the jaw muscles was mandatory.

For TMD diagnosis according to patient‐reported symptoms, the presence of facial pain or jaw‐related problems were required as follows:

Mild to very severe problems in at least three of the following six items; stiffness/fatigue of the jaw, pain in the face or jaw, pain on moving the jaw, problems opening the mouth, pain or soreness in the jaw muscles, problems yawning. On the other hand, symptoms of facial pain according to the following items: (mild‐unbearable) facial pain right now, how strong pain during the last month (maximum and average), and additionally a reported impact of facial pain on daily activities. The definition of TMD has earlier been described in detail in the article TMD in HNC patients: Clinical findings and patient‐reported symptoms by Pauli et al. (2019).

Trismus was defined as MIO ≤35 mm (Dijkstra et al., 2006). A decrease in MIO by >20% at follow‐up 6 months after oncological treatment was used as a complementary endpoint to describe reduced mouth opening capacity.

### 3.3 Ethics

The study was approved by the Regional Ethical Review Board at Gothenburg University and performed in accordance with the Declaration of Helsinki. All study subjects gave their informed consent to participate.

### 3.4 Statistical methods

Potential predictors for trismus were used in regression analysis together with patient and treatments characteristics at baseline (comorbidity, age, gender, tumor location, treatment regimen, and tumour stage (TNM) stage). Furthermore, clinical examination findings and patient‐reported symptoms at baseline were used in regression analysis in order to predict restricted mouth opening. In the regression analysis, the investigated endpoint was any occurrence of trismus during the first 6 months of follow‐up. For categorical variables *n* (%) is presented. For continuous variables mean (SD)/median (min; max)/*n* = is presented. For comparison between groups Fisher's Exact test (lowest 1‐sided *p*‐value multiplied by 2) was used for dichotomous variables, the Mantel Haenszel *χ* ^2^ test for ordered categorical variables, the *χ* ^2^ test for non‐ordered categorical variables, and the Mann–Whitney *U*‐test was used for continuous variables.

## 4 RESULTS

For this study, 89 patients with HNC were available for analysis. Six patients with trismus (MIO ≤35 mm) before oncological treatment were excluded from the analysis. Hence, 83 patients were included for the final analysis of the risk for future trismus. Mouth opening before oncological treatment varied from 37 to 68 mm with a mean (SD) of 50.5 mm (Pauli et al., 2013; van Der Molen et al., 2013). A majority of the patients (78%) had advanced tumor disease with a TNM stage of III or IV. Patient characteristics and treatment information is presented in Table 1.

**Table 1:** Patient characteristics and treatment information at baseline

	*n* = 83
Age (mean, [min‐max])	58.7 (30–77)
Gender *n* (%)	
Male	54 (65)
Female	29 (35)
Tumor location *n (%)*	
Tonsil	47 (57)
Oral cavity	6 (7)
Oropharynx	22 (27)
Salivary gland	3 (4)
Nasopharynx	5 (6)
TNM stage *n (%)*	
I	4 (5)
II	14 (17)
III	17 (21)
IV	47 (57)
Missing	1
Treatment regimen *n (%)*	
Radiotherapy	10 (12)
Radiotherapy + chemotherapy	59 (71)
Surgery + RT	11 (13)
Surgery + RCT	3 (4)
Comorbidity** *n (%)*	
No comorbidity	37 (48)
Mild comorbidity	26 (34)
Moderate comorbidity	12 (15)
Severe comorbidity	2 (3)
Missing	6

*Note:* **Comorbidity according to Adult Comorbidity Evaluation 27 (ACE‐27) classification.

### 4.1 Oncological treatment regimens

All patients in the study received external beam RT according to the local oncological guidelines in 2007–2012. The external beam RT was generally administered as accelerated fractionated RT to a total dosage of 64.6 Gy to the tumor or 68 Gy during the latter part of the study (2010–2012). Chemotherapy was generally administered as inductive cisplatin‐fluorouracil therapy before RT or concomitant cisplatin throughout the treatment course.

### 4.2 Temporomandibular dysfunction

Before the oncological treatment, 48% of the patients had clinical signs of TMD and 39% of the patients reported TMD symptoms. The corresponding figures at 6 months were 82% and 75% respectively. At the 6 months follow‐up, more than a third of the patients (35%) were examined with reduced mouth opening of >20% compared to baseline. A majority of the patients had bilateral tenderness of both the temporal and the masseter muscle on the same occasion, as per Table 2.

**Table 2:** TMD clinical examination findings for HNC patients

Clinical examination *n* (%)	Baseline *n* = 83	6 months *n* = 83
Restricted mouth opening^a^	0	14 (17)
MIO reduction >20%	NA	29 (35)
Restricted side movements^b^	16 (22) %	12 (19) %
Missing	10	20
Temporomandibular joint		
No tenderness	70 (92)	51 (76)
Unilateral tenderness	2 (3)	9 (14)
Bilateral tenderness	4 (5)	7 (10)
Missing	7	16
Temporalis anterior		
No tenderness	75 (98)	65 (93)
Unilateral tenderness	1 (1)	3 (4)
Bilateral tenderness	1 (1)	2 (3)
Missing	6	13
Temporalis insertion		
No tenderness	52 (67)	22 (31)
Unilateral tenderness	12 (16)	9 (12)
Bilateral tenderness	13 (17)	41 (57)
Missing	6	11
Masseter origin		
No tenderness	60 (78)	36 (50)
Unilateral tenderness	11 (14)	6 (8)
Bilateral tenderness	6 (8)	30 (42)
Missing	6	11
Masseter muscle		
No tenderness	59 (77)	23 (32)
Unilateral tenderness	8 (10)	11 (15)
Bilateral tenderness	10 (13)	38 (53)
Missing	6	11
TMD clinical examination	38 (48)	65 (82)

Abbreviation: HNC, head and neck cancer.

^a^ Maximal Interincisal opening ≤35 mm.

^b^ Side movement ≤6 mm.

### 4.3 Patient‐reported outcome

At the 6 months follow‐up, 42% of the study patients reported mouth opening problems. About one‐third of the patients suffered from fatigue and stiffness of the jaw as well as pain on mowing the jaw. Problems with eating solid food was reported in 50% of the patients, Table 3.

**Table 3:** Patient‐reported symptoms: For HNC patients according to Gothenburg Trismus questionnaire at baseline and at 6 months after treatment

GTQ	Baseline *n* = 83	6 months *n* = 83
	Moderate‐very severe *n* (%)	Moderate‐very severe *n* (%)
Fatigue/stiffness jaw	9 (11)	27 (33)
Pain face or jaw	5 (6)	17 (21)
Pain moving jaw	7 (8)	22 (27)
Problems opening mouth wide	7 (8)	17 (21)
Pain jaw muscles	7 (8)	17 (21)
Problem yawning	10 (12)	21 (26)
Noises from jaw	6 (7)	16 (20)
Problems eat solid food	13 (18)	38 (50)
Problems put food in mouth	8 (11)	27 (36)
Problems eat soft food	2 (3)	23 (30)
Problems bite off	2 (3)	19 (25)

	Sometimes/often/very often	Sometimes/often/very often
Clench your teeth	7 (9)	22 (27)
Press with your tongue	3 (4)	11 (14)

	Moderately/very	Moderately/very
Limitation in opening mouth	5 (6)	33 (42)

	Baseline *n* (%)	6 months *n* (%)
TMD patient‐reported symptoms	30 (39)	55 (75)

Abbreviation: HNC, head and neck cancer.

### 4.4 Risk factor models

Logistic regression was performed in order to explore which risk factors can predict the development of trismus after RT in HNC. Univariable logistic regression analysis for MIO ≤35 mm explained by characteristics at baseline (age, gender, tumor location, TNM stage, treatment regimen, comorbidity according to ACE‐27) was performed, where none of these factors were found to be significant risk factors for trismus. Analysis results showed a trend toward an increased risk for women to develop trismus and for patients with oropharyngeal tumors, albeit not significant, Table 4. In the logistic regression analysis for a decrease in MIO >20% no significant risk factors were found.

**Table 4:** Univariable logistic regression for MIO ≤35 mm explained by patient characteristics, tumor stage, and treatment regimen at baseline

Predictors	*n* missing	OR (95%CI)	*p*‐value
Age (years)	0	1.00 (0.95–1.06)	0.93
Sex (male)	0	0.44 (0.15–1.29)	0.14
Tonsil tumor	0	0.71 (0.25–2.03)	0.52
Oropharyngeal tumor	0	2.12 (0.70–6.43)	0.18
Stage	1		
III versus IV		1.30 (0.34–4.94)	0.70
II versus IV		1.69 (0.43–6.64)	0.45
I versus IV		lim(OR) = 0	
Treatment regimen	0		
Radiotherapy + chemotherapy versus radiotherapy		1.36 (0.26–7.15)	0.71
Surgery + radiotherapy versus radiotherapy		0.40 (0.03–5.25)	0.49
Surgery + radiotherapy + chemotherapy versus radiotherapy		lim(OR) = 0	
Comorbidity	6		
Mild comorbidity versus no comorbidity		0.35 (0.09–1.44)	0.15
Moderate comorbidity versus no comorbidity		1.93 (0.50–7.50)	0.34
Severe comorbidity versus no comorbidity		lim(OR) = 0	

Abbreviation: MIO, maximal interincisal opening.

### 4.5 TMD as a risk factor for trismus

When analyzing TMD prior RT as risk factor for restricted mouth opening after RT, both according to clinical examination findings and using patient‐reported outcome in regression analysis, a tendency toward an increased risk of reduced mouth opening was seen but was not statistically significant, OR 4.7 (95% CI 1–22, *p* = 0.054) Table 5. Similarly, for single clinical examination findings, unilateral muscular tenderness in the memporal muscle insertion, the increased risk of restricted mouth opening was OR 3.9 (95% CI 1.0–15.5, *p* = 0.051) Table 6.

**Table 5:** Univariable logistic regression for MIO ≤35 mm explained by clinical examination findings

Baseline variable	*n* missing	OR (95%CI)	*p*‐value
Side movement (mm)	10	0.84 (0.66–1.06)	0.15
Reduced horizontal mandibular mobility	10	1.57 (0.42–5.87)	0.51
Side movement (mm)	10	0.96 (0.78–1.18)	0.71
Reduced horizontal mandibular mobility	10	0.88 (0.21–3.59)	0.85
Muscle tenderness summation score	6	0.99 (0.73–1.35)	0.96
Temporalis anterior	6	1.00	
Unilateral tenderness		0.00 (0.00–infinity)	0.98
Bilateral tenderness			
Temporalis insertion	6	1.00	
Unilateral tenderness		3.93 (1.00–15.50)	0.051
Bilateral tenderness		1.00 (0.19–5.39)	1.00
Masseter origin	6	1.00	
Unilateral tenderness		0.36 (0.04–3.09)	0.35
Bilateral tenderness		0.72 (0.08–6.75)	0.78
Masseter muscle	6	1.00	
Unilateral tenderness		0.56 (0.06–4.99)	0.60
Bilateral tenderness		0.98 (0.18–5.22)	0.98
Temporomandibular joint	7	1.00	
Unilateral tenderness		0.00 (0.00–infinity)	0.99
Bilateral tenderness		4.38 (0.56–34.07)	

Abbreviation: MIO, maximal interincisal opening.

**Table 6:** Univariable logistic regression for MIO ≤35 mm explained by patient‐reported outcome (GTQ) at baseline and summarized score for TMD subjective and objective

Baseline variable	*n* missing	OR (95% CI)	*p*‐value
TMD subjective + objective	7	4.67 (0.97–22.41)	0.054
TMD subjective	6	1.48 (0.48–4.63)	0.50
TMD objective	3	1.14 (0.40–3.25)	0.81
Fatigue/stiffness jaw	0	1.97 (0.44–8.79)	0.38
Pain face or jaw	1	2.54 (0.39–16.52)	0.33
Pain moving jaw	0	5.90 (1.19–29.40)	0.030
Problems opening mouth wide	0	1.97 (0.44–8.79)	0.38
Pain jaw muscles	0	5.90 (1.19–29.40)	0.030
Problem yawning	0	2.81 (0.70–11.31)	0.15
Noises from jaw	0	1.91 (0.32–11.35)	0.48
Problems eat solid food	9	0.92 (0.22–3.79)	0.91
Problems put food in mouth	9	0.41 (0.05–3.59)	0.42
Problems eat soft food	9	3.24 (0.19–54.53)	0.42
Problems bite off	9	3.24 (0.19–54.53)	0.42
Clench your teeth	1	1.48 (0.26–8.32)	0.66
Press with your tongue	2	1.79 (0.15–21.00)	0.64
Limitation in opening mouth	3	0.85 (0.09–8.15)	0.89
GTQ domains			
Jaw related problems	0	1.02 (1.00–1.05)	0.072
Eating limitation	9	1.00 (0.97–1.03)	0.88
Muscular tension	1	1.03 (0.99–1.07)	0.14

Abbreviations: GTQ, Gothenburg Trismus Questionnaire; MIO, maximal interincisal opening.

In terms of TMD according to the patient‐reported outcome, two of the items of the GTQ were found to be related to a statistically significant increased risk of restricted mouth opening after RT. “During the last week, have you felt: Pain on moving the jaw?” (OR 5.9 [95% CI 1.2–29.4, *p* = 0.030]) and “During the last week, have you felt pain and tenderness in the muscles of mastication?” (OR 5.90 [95% CI 1.19–29.40, *p* = 0.030]) Table 6. None of the investigated risk factors showed a strong predictive ability and hence no more advanced logistical regression models were created.

## 5 DISCUSSION

In this prospective study investigating the impact of TMD symptoms as a risk factor for developing radiation‐induced trismus, it was found that patients with HNC who reported pain in the jaw muscles and pain when moving the jaw prior to RT are at higher risk for developing radiation‐induced trismus after oncological treatment.

None of the other clinical examination findings or investigated parameters were found to be strong predictors for trismus in our dataset.

A tendency toward a higher risk of trismus in women was seen and has earlier been highlighted as a risk factor for trismus. This is probably due to women having a habitually smaller mouth opening in general, thus predisposing them to an increased risk of fulfilling the trismus criteria (MIO ≤35 mm) (Wetzels et al., 2014).

For the study patients, as well as in the general population, TMD symptoms and clinical signs of TMD are very common and are often the result of longstanding muscle hyperactivity or parafunctional activity of the masticatory system (Okeson, 2013). It is of course difficult to distinguish if the TMD symptoms and clinical signs that the HNC patients presents with prior to RT are related to the tumor or increased muscle tension due to the mental stress that the HNC diagnoses induced, or are a result of more chronic symptomatology. Regardless, it is very clear that the masticatory structures are affected by the RT and consequently, the patients experience problems with opening the mouth, pain, fatigue, and stiffness when moving the jaw. In the same way tenderness of the jaw muscles (particularly the masseter and the insertion of temporal muscles) as well as a clear decrease in mouth opening capacity can be found upon clinical examination.

Orofacial pain and problems with mouth opening after HNC are related to an increased risk of malnutrition and can have a negative impact on the patient's health‐related quality of life (Johnson et al., 2015; Lee et al., 2012; Weber et al., 2010). It is therefore important to address and optimize the patient's pain treatment as much as possible. Both pharmacological and physiotherapeutic treatment measures should be considered. For the treatment of TMD and trismus, jaw exercise therapy has been reported to improve mouth‐opening capacity and reduce orofacial pain (Kamstra et al., 2013; Makino et al., 2018; Pauli et al., 2016).

This study shows that it is of great importance to identify patients who suffer from orofacial pain prior to the oncological treatment since they seem to be at risk of being more severely affected post‐RT in terms of developing radiation‐induced trismus. This condition is associated with pain, problems with teeth, and oral health as well as a risk of malnutrition. We found no single clinical sign that could predict trismus but rather the patient's own reported symptoms of orofacial pain before oncological treatment should be considered a red flag for radiation‐induced trismus. The results are a reminder of how important patient‐reported outcomes are both in clinical trials and in health care in general.

A study limitation was the relatively small sample size. A more robust risk factor model for trismus could probably have been developed in a larger cohort of patients. Moreover, since the studied endpoints were relatively common, such as “reduction in mouth opening >20%,” (observed in as many as 35% of the patients) the statistical demands on sample size rises.

## 6 CONCLUSION

TMD is common amongst HNC patients. Patients who suffer from pain in the jaw muscles and pain when moving the jaw before start of oncological treatment are at higher risk of radiation‐induced trismus after 6 months. Clinicians should strive to optimize the patients' pain treatment and oral health before, during, and after RT.

## CONFLICT OF INTEREST

The authors have no other funding, financial relationships, or conflicts of interest to disclose.

## AUTHOR CONTRIBUTIONS

**Nina Pauli**: Conceived and designed the analysis; wrote the paper. **Bodil Fagerberg Mohlin**: Collected the data; contributed data or analysis tools. **Christina Mejersjö**: Conceived and designed the analysis; other contribution (critical review of manuscript); **Caterina Finizia**: Conceived and designed the analysis; contributed data or analysis tools; other contribution (critical review of manuscript).

## Data Availability

The data that support the findings of this study are available from the corresponding author, Nina Pauli, upon reasonable request.
